# A Rapid, Multiplexed, High-Throughput Flow-Through Membrane Immunoassay: A Convenient Alternative to ELISA

**DOI:** 10.3390/diagnostics3020244

**Published:** 2013-04-02

**Authors:** Sujatha Ramachandran, Mitra Singhal, Katherine G. McKenzie, Jennifer L. Osborn, Amit Arjyal, Sabina Dongol, Stephen G. Baker, Buddha Basnyat, Jeremy Farrar, Christiane Dolecek, Gonzalo J. Domingo, Paul Yager, Barry Lutz

**Affiliations:** 1 Department of Bioengineering, University of Washington, Seattle, WA 98195, USA; E-Mails: k.g.mckenzie@gmail.com (K.G.M.); jen.l.osborn@gmail.com (J.L.O.); yagerp@uw.edu (P.Y.); blutz@u.washington.edu (B.L.); 2 PATH, Seattle, WA 98121, USA; E-Mails: msinghal@path.org (M.S.); gdomingo@path.org (G.J.D.); 3 Oxford University Clinical Research Unit-Patan Academy of Health Sciences, Kathmandu, Nepal; E-Mails: amitarjyal@yahoo.com (A.A.); dongolsabina@yahoo.com (S.D.); rishibas@wlink.com.np (B.B.); 4 The Hospital for Tropical Diseases, Wellcome Trust Major Overseas Programme, Oxford University Clinical Research Unit, Ho Chi Minh City Q5, Vietnam; E-Mails: sbaker@oucru.org (S.G.B.); jfarrar@oucru.org (J.F.); cdolecek@oucru.org (C.D.)

**Keywords:** flow-through membrane immunoassay (FMIA), enzyme-linked immunosorbent assay (ELISA), multiplex, indirect IgM assay, *Salmonella enterica *serovar Typhi, typhoid, serodiagnosis, low resource setting

## Abstract

This paper describes a rapid, high-throughput flow-through membrane immunoassay (FMIA) platform. A nitrocellulose membrane was spotted in an array format with multiple capture and control reagents for each sample detection area, and assay steps were carried out by sequential aspiration of sample and reagents through each detection area using a 96-well vacuum manifold. The FMIA provides an alternate assay format with several advantages over ELISA. The high surface area of the membrane permits high label concentration using gold labels, and the small pores and vacuum control provide rapid diffusion to reduce total assay time to ~30 min. All reagents used in the FMIA are compatible with dry storage without refrigeration. The results appear as colored spots on the membrane that can be quantified using a flatbed scanner. We demonstrate the platform for detection of IgM specific to lipopolysaccharides (LPS) derived from *Salmonella *Typhi. The FMIA format provides analytical results comparable to ELISA in less time, provides integrated assay controls, and allows compensation for specimen-to-specimen variability in background, which is a particular challenge for IgM assays.

## 1. Introduction

The enzyme-linked immunosorbent assay (ELISA) is widely used as a diagnostic test in laboratories, but it has many limitations. ELISA takes several hours or more to perform, uses reagents that require cold storage, and normally requires a plate reader to measure the results. Additionally, ELISA as typically performed requires separate wells for each analyte tested, and additional wells for assay controls and background measurements, thus reducing throughput and increasing opportunity for well-to-well errors. There is a general need for diagnostic methods capable of rapid high-throughput testing of multiple analytes; this is particularly true for low-resource settings [1,2]. 

**Figure 1:**
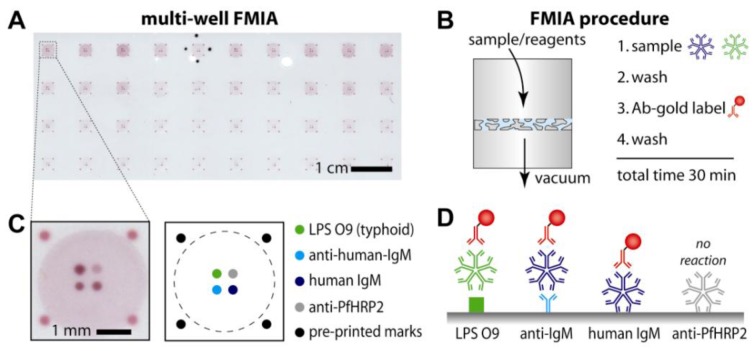
Flow-through membrane immunoassay (FMIA) format. (**A**) Image of the multi-well assay membrane (96 well format, 10 × 4 wells shown). (**B**) Sequence of FMIA steps. An array of sample detection regions was defined by a BioDot 96-well microfiltration apparatus; the flow-through region for each sample (a “well”) was defined by an open-bottom well above the membrane and a corresponding hole in a rubber gasket below the membrane (see Materials and Methods). (**C**) Schematic of the capture spots and control spots on the nitrocellulose membrane for a single well. Protein-coated gold (40 nm) was spotted on the outer edges as markers for alignment of the capture membrane on the vacuum manifold. The four innermost spots are assay and control spots (spot spacing is 500 µm center to center). (**D**) Schematic of the indirect anti-LPS IgM assay and control assays.

Here, we present a rapid high-throughput flow-through membrane immunoassay (FMIA) platform as an alternative to ELISA. A schematic of the FMIA format is depicted in Figure 1. The FMIA is carried out using a 96-well vacuum plate to draw sample and reagents through a nitrocellulose membrane spotted with capture molecules; gold nanoparticle-labeled secondary antibodies (identical to those used in lateral flow tests) are used to provide visible assay signals. The FMIA provides rapid results (<30 min), requires fewer user steps than ELISA, provides multiple assay results (including controls) for each sample, uses reagents that can be stored in stable dry form, and generates visible spots that can be quantified by a camera or a flatbed scanner [3,4,5]. The FMIA format was originally developed as an in-house tool in the development of a point-of-care immunoassay system for the developing world—the DxBox project supported by The Bill & Melinda Gates Foundation’s Grand Challenges in Global Health Initiative [2,3]. However, the benchtop assay format proved sufficiently convenient compared to ELISA that we present it here as a stand-alone assay. 

Dot-blot immunoassays using porous nitrocellulose membranes have been reported for diagnosis of various diseases [6]. Cardosa *et al.* reported a MAC DOT format in which the capture reagent was spotted onto nitrocellulose in an array, and the assay was carried out by cutting out individual pieces and incubating them separately with detection reagents [7]. Cardona-Castro *et al. *used a microfiltration apparatus to perform an immunoenzymatic dot-blot test on a nitrocellulose membrane [8]. However, in this method the sample and reagents flowed through the membrane by gravity alone, which resulted in long assay times. A similar technique was used by Van Vooren *et al.* in a comparative study to ELISA, but the apparatus was disassembled to remove the membrane for enzymatic detection [9]. Another technique reported by Zalis and Jaffe used a modified flat-bottom 96-well microtiter plate to perform a dot-blot assay using clamped filter paper [10]. These methods produce colored spots that can be detected without a plate reader, but as reported they give qualitative results, require long assay times, and do not integrate controls for each sample. The FMIA method described here shares many features with the dot-blot assays but addresses limitations in speed, throughput, and quantification that make it appealing as an alternative for ELISA.

The purpose of this paper is to describe the methods used in the FMIA and to evaluate the analytical performance compared to ELISA for an example serology assay. For this demonstration, we chose detection of IgM antibodies to *Salmonella enterica *serovar Typhi (hereafter, *Salmonella* Typhi), which causes typhoid fever [11,12,13,14,15]. The FMIA also included measurement of an endogenous control, a process control, and non-specific background for each sample. We tested a panel of patient samples by FMIA and ELISA, and we used the same capture antigen and detection antibody in both formats to allow analytical comparison without influence of reagent selection. Thus, our intention was not to assess clinical performance nor to validate the specific assay, but rather to compare features and performance of the two platforms. The FMIA allows testing for multiple targets from a single sample and integrates assay controls in each sample detection region, and it provides analytical results comparable to ELISA in less time.

## 2. Materials and Methods

### 2.1. Test Samples

Since a purified analyte was not available for creating samples, existing patient specimens were used for platform testing. Specimens had been collected from patients presenting to Patan Hospital in Kathmandu, Nepal with typhoid fever (Current Controlled Trials ISRCTN 53258327; human subjects approvals: OXTREC 002-6 and HS 393); a second sample was collected from these patients one week later, as is customary for typhoid serological diagnosis (designated here as “*Day 1*” samples and “*Day 8*” samples). Plasma was separated from blood cells by centrifugation and stored at −20 °C until use. Samples were tested by microbiological culture and ELISA. Specimens were classified as positive if they had confirmed *Salmonella* Typhi infection by microbiological culture and an increase in IgM titers were observed between Day1 and Day 8. Specimens were classified as negative if *Salmonella *Typhi was not detected by microbiological culture, and the IgM signal was below cut-off in both Day 1 and Day 8 for the starting titer. The test results were used to select a sample set that included positive samples with a range of assay signals and negative samples, but classification as positive and negative was not used in the analytical comparison presented here.

### 2.2. Conventional ELISA

#### 2.2.1. Preparation of ELISA Capture Plates for the Indirect IgM Assay

Immulon II HB plates (Thermo Fisher Scientific Inc., Waltham, MA, USA) were coated with purified lipopolysaccharide (LPS) O9 antigen specific to *Salmonella *Typhi (Sigma Aldrich, St Louis, MO, USA) in phosphate buffered saline (PBS, pH 7.4). Fifty microliters of LPS O9 at 1 µg/mL in each well was incubated overnight at 4 °C. Coated plates were washed once by PBS with 0.05% Tween 20 (PBST), blocked with 5% non-fat dried milk in PBS for two hours at 25 °C, washed with PBST, and sealed with desiccant for dry storage at −20 °C until use.

#### 2.2.2. Sample Preparation and IgG “Mop-Up”

The indirect IgM assay is normally performed after IgG sequestration (“mop-up”) and sample dilution. The “mop-up” process is commonly used in the indirect IgM format to reduce interference from disease-specific IgG and rheumatoid factor (false negatives due to IgG binding competition with IgM, false positives due to IgG-rheumatoid factor complexes that would be detected by the IgM detection reagent). The sample dilution buffer BUF 38A (ABD Serotech, Raleigh, NC, USA) was diluted 1:8 from stock with PBS, and the mop-up reagent was polyclonal goat anti-human IgG, Fc specific (International Immunology Corp., Murrieta, CA, USA) at a dilution of 1:64. This treatment does not remove IgG from the sample, but its effect is to reduce binding of IgG to the capture antigen (putatively due to formation of IgG aggregates) [16,17]. Plasma samples were thawed on ice, and a titration series for each sample was created by two-fold dilutions of the plasma in this solution (final sample dilutions 1:100–1:800). 

#### 2.2.3. ELISA Procedure for the Indirect IgM Assay

ELISA plates were equilibrated to room temperature and 50 µL of prepared sample (diluent and “mop up” reagent) was incubated in each well for 1 h at 25 °C, followed by washing four times with PBST. The detection reagent was prepared by diluting HRP-conjugated anti-human-IgM antibody (horseradish peroxidase-conjugated AffiniPure Goat anti-human-IgM, Fc5µ fragment specific; Jackson ImmunoResearch Laboratories Inc., West Grove, PA, USA) 1:10,000 in stock BUF 38A. 50 microliters of the diluted detection reagent was incubated in each well for 1 h at 25 °C, and then the plates were washed four times with PBST. The plates were developed by adding 50 µL of pre-warmed 3,3',5,5' tetramethyl benzidine (TMB) substrate (Sigma Aldrich, St Louis, MO, USA) to each well and incubating in the dark for 10 min at 25 °C. Color development was stopped by addition of 50 µL of 4 N sulfuric acid to each well. The colorimetric signal was measured in Spectramax ELISA plate reader set at an absorbance of 450 nm with a reference wavelength of 510 nm. 

### 2.3. Flow-Through Membrane Immunoassay (FMIA)

The FMIA used the same capture antigen (purified LPS O9) and the same detection antibody (AffiniPure Goat anti-human-IgM, Fc5µ fragment specific) used in the ELISA format. The detection antibody was conjugated to gold nanoparticles instead of the horseradish-peroxidase (HRP) used in ELISA, and buffers and blocking agents were slightly different (see below). Additional control assays were included in each sample detection region in the FMIA.

#### 2.3.1. Preparation of FMIA Capture Membrane for the Indirect IgM Assay

Nitrocellulose membranes with 0.45 µm pore size (Whatman^®^ Protran^®^ BA85 from MIDSCI, St. Louis, MO, USA) were spotted with capture reagents using a Scienion Biodot spotter (80 droplets per spot, ~30 nanoliters total volume per spot) in a pattern corresponding to a standard 96-well plate format (Figure 1(A)). Each sample detection region was spotted with purified LPS O9 at 1.0 mg/mL in PBS for the typhoid IgM assay, as well as assay controls created by spotting anti-human-IgM at 0.5 mg/mL in PBS (AffiniPure Goat anti-human-IgM, Fc5µ fragment specific; Jackson ImmunoResearch Laboratories Inc., West Grove, PA, USA), purified human IgM at 0.25 mg/mL in PBS (Sigma Aldrich, St Louis, MO, USA), and anti-*Plasmodium falciparum* HRP2 (*Pf*HRP2) at 0.5 mg/mL in PBS (Immunology Consultant Laboratories Inc., Newberg, OR, USA). The membranes were immersed in membrane blocking solution (Invitrogen, Carlsbad, CA, USA) for 1 h at 25 °C, dried, and stored in a desiccator at 25 °C until use. 

#### 2.3.2. Sample Preparation and IgG Removal

In the IgG “mop-up” used for conventional ELISA, IgG is sequestered but not removed (it remains in the sample), and we found that it led to high background when used in the FMIA (presumably due to entrapment of aggregated IgG). We previously described a bead-based method for removing IgG from the sample prior to IgM indirect assays, and we implemented the method in a benchtop assay and microfluidic cards using dry-stored beads [18]. Here, plasma samples (5 µL) were diluted in 432.5 µL Tris-buffered saline containing 0.1% Tween 20 (TBST, pH 8.2) and mixed with 125 µL UltraLink^®^ protein-G-coated beads (Thermo Fisher Scientific Inc., Waltham, MA, USA). The protein-G-coated beads selectively bind IgG antibodies without significant removal of the target IgM antibodies; beads coated with anti-human-IgG would be a suitable alternative. The mixture was vortex-mixed for 5 min, and beads were removed from the sample by centrifugation through a spin column at 1,000 ×*g* for 1 min (Thermo Fisher Scientific Inc., Waltham, MA, USA). This procedure gave a 1:100 dilution of the samples. A series of two-fold dilutions of the samples were made with TBST (final sample dilutions 1:100–1:3,200). Plasma was used as the sample here; using whole blood as the input would require cell separation by centrifugation or filtration prior to the IgG removal step. We have demonstrated detection from whole blood samples in a microfluidic card format that included a plasma separation membrane to allow input of whole blood to the device [19].

#### 2.3.3. FMIA Procedure for the Indirect IgM Assay

A microfiltration apparatus was used to define wells over each sample detection region (Bio-Dot^®^ Microfiltration apparatus from Bio-Rad, Hercules, CA, USA). The system is designed to sandwich a membrane between a plastic frame and rubber gasket with 96 holes (below the membrane) and a plastic frame with 96 open-bottom wells (above the membrane). Fluids added to the wells were pulled through the membrane to a waste chamber by a vacuum source connected to the lower frame. The spotted membrane was pre-wet with PBS so that sample would not wick into the spaces between wells, and the two frames were tightened by thumbscrews to prevent leakage between wells. The components described above are all part of the BioRad device as purchased. The membrane was aligned using pre-printed gold reference markers so that the capture spots aligned with the flow-through holes on the gasket. The apparatus was connected to house vacuum regulated by a vacuum regulator and outfitted with a vacuum gauge. For the assay, 200 µL of IgG-depleted diluted sample was applied to each well, incubated for 4 min, and pulled through the membrane under vacuum control (2.5 kPa). The membranes were then washed twice with 600 µL of TBST under vacuum control (7.5 kPa). The detection reagent (denoted Ab-gold) was prepared by diluting gold-conjugated anti-human-IgM antibody (custom conjugation from Arista Biologicals, Allentown, PA, USA, using AffiniPure Goat anti-human-IgM, Fc5µ fragment specific from Jackson ImmunoResearch Laboratories Inc., West Grove, PA, USA) in Tris-buffered saline (TBS) containing 1% BSA. One hundred microliters of diluted detection reagent was incubated in each well for 4 min at 25 °C, then the reagent was pulled through under vacuum control (2.5 kPa), and the membrane was washed with 600 µL of TBST.

#### 2.3.4. Image Capture and Quantification

Assay membranes were imaged with a conventional office flatbed scanner (ScanMaker i900, MicroTek International, Inc., Cerritos, CA, USA) in 48-bit RGB mode at a resolution of 600 ppi. The spot intensities were quantified using ImageJ [20] by measuring mean green-channel intensity of each assay spot and a background region within each well; circular regions of interest for intensity measurements were approximately one-half the full spot size. Background signal representing non-specific binding was averaged from three spots in the un-spotted region of the flow-through area (region that was not spotted with capture molecules but was blocked). The background was quite uniform throughout the unspotted region. The reflectance optical density (OD) was calculated as FMIA OD = log(*white*/*assay spot*), where “*white*” was the pixel intensity of a wetted membrane outside the assay well. The assay signal was reported as the assay spot OD minus the background OD; background-subtracted FMIA OD = log(*background*/*assay spot*) [4,21]. 

## 3. Results

### 3.1. The Flow-Through Membrane Immunoassay (FMIA) Format

Figure 1 illustrates the FMIA format and assay procedure. Multiple capture spots for the assay and controls were patterned on nitrocellulose membranes within each well area on a 96-well grid pattern (Figure 1(A)); spotted membranes were stored dry at room temperature until needed. For the assay, the sample, wash buffer, and colorimetric detection label were applied manually and pulled through the membrane by controlled vacuum using a 96-well vacuum manifold (Figure 1(B)). The total assay time was 30 min, and the assay result was a pattern of visible spots (Figure 1(C)).

Figure 1(C) shows the arrangement of capture molecules spotted within each sample flow-through area, and Figure 1(D) shows the component stack for each assay spot. For the serology assay, we adopted the indirect format that uses spotted antigens to capture disease-specific IgM antibodies from the sample. IgM assays are commonly used to identify current (as opposed to past) infections and the indirect format allows multiplexing analytes within a single well. Further, since specificity (and spatial location) is provided by the spotted antigen, all analytes can be detected using a single detection reagent that binds human IgM. 

Lipopolysaccharide (LPS) O9 antigen was spotted to capture typhoid IgM antibodies. Anti-human-IgM antibody was spotted to capture IgM (disease-specific as well as non-disease-specific) which served as an endogenous control to confirm that a blood sample was used, and purified human IgM was spotted as a process control to confirm correct application of the anti-human-IgM detection reagent. An unrelated antibody was spotted for a potential second assay (malaria antigen assay; this assay spot was not used in the analysis presented here). The endogenous control and process control were used qualitatively to verify successful application of sample and reagents, but signals were not used in the quantitative analysis. The unspotted region of the membrane served as a control for non-specific binding; the background signal was subtracted from assay signals in the analysis. Because controls were integrated within each well, 96 individual samples could be processed in a single run. 

### 3.2. Representative Images from the FMIA

**Figure 2:**
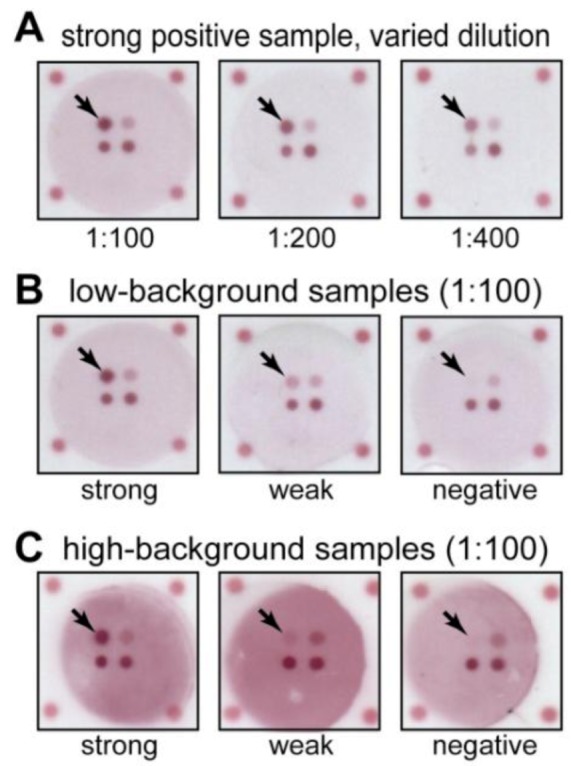
Example images from the FMIA format for indirect anti-LPS IgM detection with integrated control assays. Designations of strong, weak, and negative are based on the ELISA results. The anti-LPS IgM capture spots are indicated by arrows. (**A**) Example of a strong sample at different sample dilutions. The typhoid-specific IgM spot (upper left in each well) and endogenous IgM control spot (lower left in each well) respond to sample dilution, but the process control signal (lower right in each well) remains constant. (**B**) Examples of strong, weak, and negative samples with low background tested at a single dilution (1:100). The typhoid-specific IgM spot intensities mirror the results from ELISA, while control spots (lower in each well) are consistent between samples. (**C**) Examples of strong, weak, and negative samples that showed high background in non-spotted regions.

**Figure S1:**
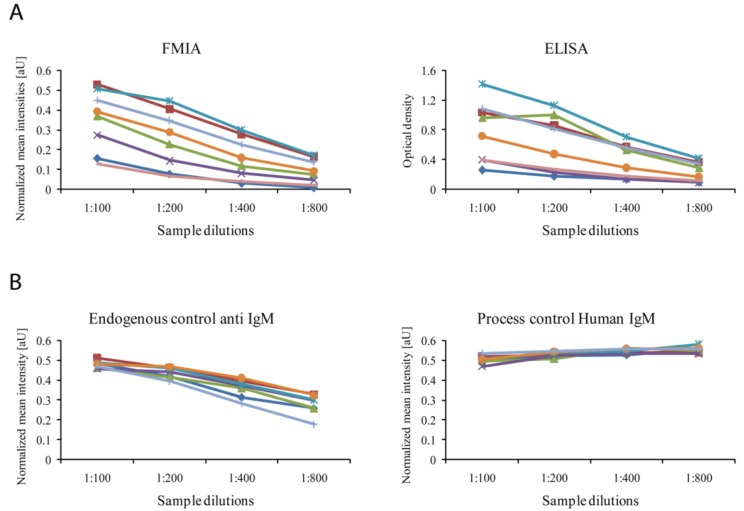
(**A**) Sample dilution series for samples tested by FMIA and ELISA. The subset of samples shown was chosen from samples spanning strong to weak signals in ELISA. The FMIA and ELISA show similar responses across the dilution series. (**B**) Endogenous controls and process controls for the same samples as measured in the FMIA across the full dilution series. The endogenous control (detection of total IgM antibody in the sample) decreased in intensity with dilution as expected, while the process control (capture of the Ab-gold detection reagent by spotted human IgM) was constant across all dilutions, as expected.

Figure 2(A) shows FMIA images for a strong positive sample tested over a range of sample dilutions. The anti-LPS IgM spots (upper left in each well) decreased in intensity with sample dilution as expected. The endogenous control spots (lower left in each well) also decreased in intensity with dilution as expected, but they remained detectable over a range of dilutions. The process control spots (lower right in each well) confirmed that Ab-gold was applied properly, and it showed a constant intensity for all sample dilutions as expected. Together, these control spots can be used to reject results due to procedural errors. Supplementary Figure S1 provides the FMIA and ELISA signal data for a dilution series performed on a subset of samples, as well as providing data for the process control and endogenous control signals from the FMIA for the dilution series. The 1:100 dilution used the largest portion of the dynamic range for this sample set, but all dilutions provide measurable distribution of intensities (*i.e.*, detectable signal and no saturation). FMIA and ELISA show similar responses to dilution, and the controls show the expected response for all samples. A non-specific signal on the upper right spots is relatively constant for all dilutions due to cross reactivity of the Ab-gold detection reagent (anti-human IgM) to the spotted anti-*Pf*HRP2 IgM antibodies (this spot was not used in the analysis presented here). 

**Figure S2:**
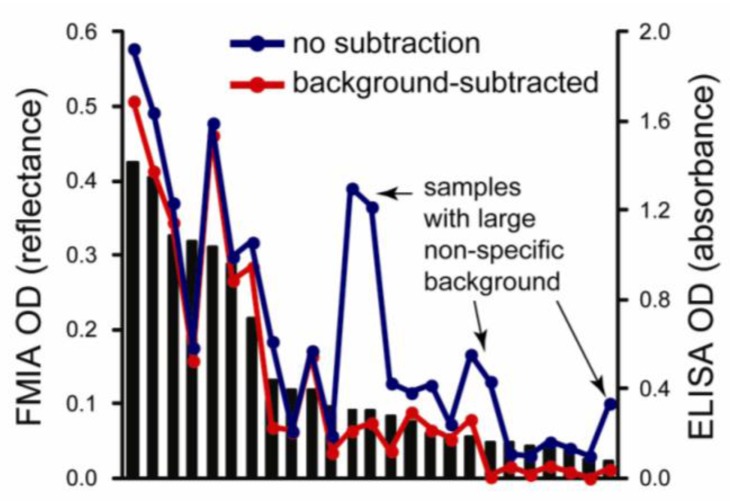
Impact of background subtraction in the FMIA. The FMIA signals presented in Figure 3 included subtraction of background signal from unspotted (but blocked) regions of the membrane for each sample. The plot below reproduces the data from Figure 3 for FMIA (red) and ELISA (black bars) with additional overlay of FMIA signals without background subtraction (blue). If background subtraction was not performed, several samples deviated from ELISA due to large non-specific binding. Background subtraction in the FMIA accounted well for effects of non-specific binding.

Figure 2(B,C) show example FMIA images for samples identified as strong positive, weak positive, and negative by ELISA. Figure 2(B) shows images for samples that had low non-specific background in the FMIA, and Figure 2(C) shows images for samples that had high non-specific background in the FMIA. Qualitatively, the anti-LPS IgM spots (upper left in each well) remained detectable above the background, even for samples with high non-specific background and weak signal (Figure 2(C), middle). Non-specific binding of IgM antibodies is a common problem in the indirect assay format; we have confirmed that elevated IgM concentration is one cause of background in the FMIA format (unpublished results). However, the ability to measure background signal in the same well as the assay allows signal to be corrected for non-specific binding. We routinely use local background subtraction to quantify spot intensities from FMIA images [3,4,5,19,22]. For the quantitative analysis presented below, we subtracted the local background signal within each well from the assay spot signals to account for non-specific binding. For ELISA, the corresponding control would require running the sample in a separate well without capture antigen, and this control was not performed for ELISA results. Non-specific binding may be larger in the FMIA format, and background subtraction helped reduce its effects (Supplementary Figure S2 plots background signals for all samples tested). For all samples, the spot intensities for endogenous and process controls (lower spots in each well) showed measurable signal and confirmed successful tests.

### 3.3. Comparison between FMIA and ELISA

For characterization of FMIA analytical performance, we selected a panel of 48 samples (24 paired *Day 1* and *Day 8*) that spanned the range of low to high ELISA signals (including negative samples). All 48 samples were tested by the FMIA and ELISA, and a subset of samples was tested in duplicate on both platforms. The same capture antigen and detection antibody were used in FMIA and ELISA to reduce variation caused by reagent selection. The purpose of this comparison was not to assess clinical performance, but rather to evaluate analytical performance of the FMIA format.

**Figure 3:**
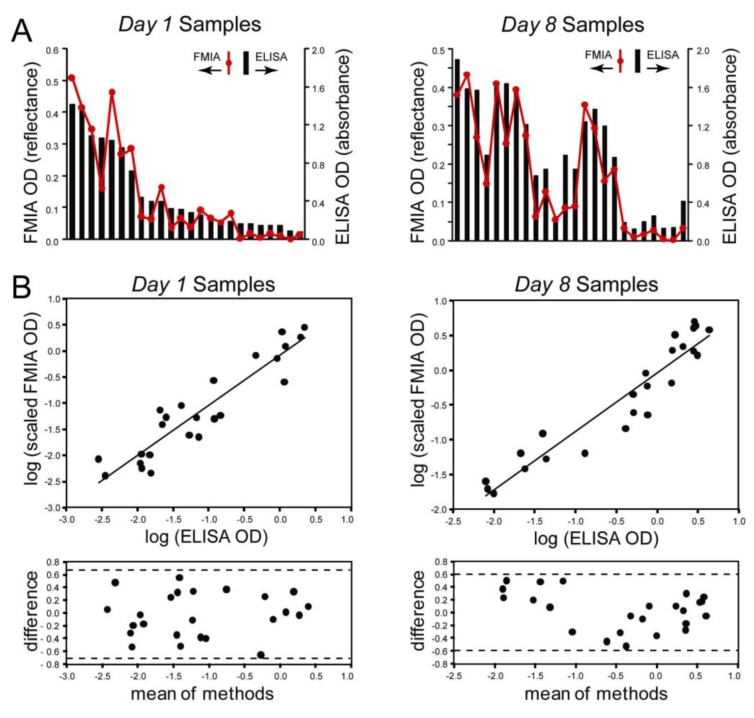
Quantitative comparison of ELISA and the FMIA format for a panel of 24 **paired** patient samples collected on the first visit from patients presenting with fever (*Day 1*) and one week later (*Day 8*). (**A**) The bars 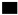 represent optical density (OD) from ELISA (absorbance measurements), and the line plots 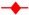 represent the background subtracted OD from the FMIA (reflectance measurements). (**B**) Log-log correlation plot of scaled FMIA OD *versus* ELISA OD for all samples reported in Panel A. The lower plots in Panel B show analysis of error distribution across the measured range (Bland-Altman analysis on scaled FMIA data). The error is well distributed and falls within the limits of agreement (dashed lines; 1.96 times standard deviation of errors).

**Figure S3:**
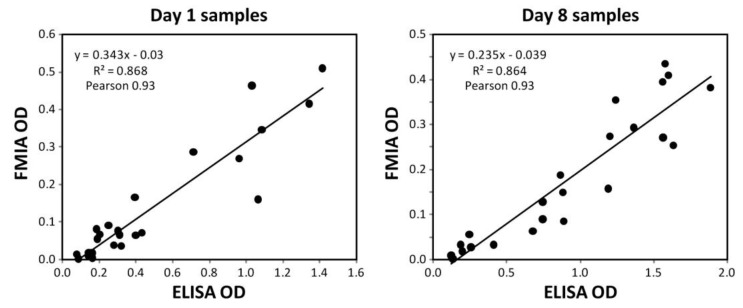
Correlation plots for raw FMIA OD and raw ELISA OD data for *Day 1* and *Day 8* samples. Pearson correlation coefficients were 0.93 for *Day 1* and *Day 8* data sets. The increasing scatter at higher assay signals is expected for many assays; the error analysis in Figure 3 confirms that error scales with signal (*i.e.*, constant CV; this is treated by applying the log-transformation in Figure 3). The best-fit correlation lines shown here were used to perform the linear scaling applied in Figure 3. *Day 1* scaling resulting in Figure 3 data: scaled FMIA = (FMIA OD − 0.030) × 0.343; coefficient of determination 0.868. *Day 8* scaling resulting in Figure 3 data: scaled FMIA = (FMIA OD − 0.039) × 0.235; coefficient of determination 0.864. Figure 3 in the text shows the linearly-scaled, log-transformed data; the plots below show the raw data.

Figure 3 compares ELISA to the FMIA for *Day 1* and *Day 8* samples tested at a single dilution (1:100). Figure 3(A) plots raw ELISA OD (bars) and background-subtracted FMIA OD (red); the paired samples are presented in the same order in the *Day 1* and *Day 8* plots. In the *Day 8* samples, a subset of patients show a net increase in assay signal as expected. Correlation of FMIA OD to ELISA OD for *Day 1* and *Day 8* data gave Pearson correlation coefficients of 0.93 for both data sets (Supplementary Figure S3).

Figure 3(B) plots the log-transformed correlation between scaled FMIA OD and ELISA OD based on the data from Figure 3(A). The FMIA data was linearly scaled to account for expected differences in OD scales for the two methods (absorbance OD for ELISA, reflectance OD for FMIA); a log transformation was applied to account for the observed scaling of error with assay signal. Figure 3(B) (lower) shows that the log-transformed error is well-distributed within the limits of agreement (dashed lines). 

**Figure S4:**
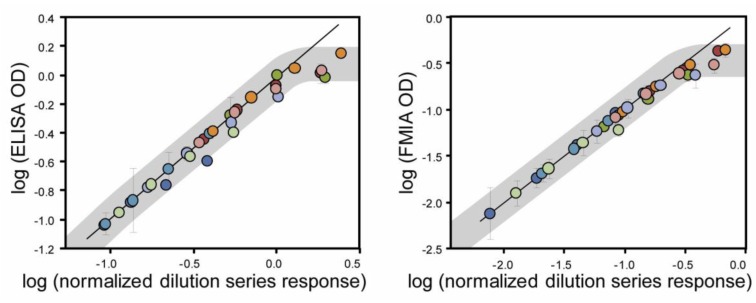
Comparison of sample dilution series measured by ELISA and FMIA. Samples were serially-diluted (1:100, 1:200, 1:400, and 1:800) and tested by ELISA and FMIA; each set of colored data points represents the dilution series for a given sample tested by both methods. There is no purified source of the target analyte (LPS-specific human IgM antibody), so creating a quantitative response curve by conventional sample spiking was not possible. Because the analyte concentrations for these patient samples are not known, the response curves for each sample dilution series were normalized to fall on a single response curve to estimate the linear response range from patient samples. Specifically, the linear portion of the dilution series for each patient sample (data points of a given color) was fit to a line, and the data points for that sample were scaled by the slope of the response curve to collapse the data on a common response curve. The normalization has no effect on the shape of individual response curves. Thus, the concentration scale is arbitrary, but it gives a qualitative indication of the linear range for the two formats. Each color represents the dilution series for an individual sample, and error bars are for two replicates performed on each sample at each dilution. Saturation at high signals is qualitatively indicated by the gray trace. The apparent linear range is similar for ELISA and FMIA.

The inter-assay *%CV’s* for FMIA and ELISA were estimated from a subset of samples tested at 1:100 dilution on different days. For FMIA, eight patient samples were tested on different days; the *%CV* for each sample was calculated, and the values were averaged. For ELISA, a positive control sample was tested in sixteen plates on different days; the *%CV* for the full set was calculated. The estimated inter-assay *%CV’s* were 15% for FMIA (*n* = 8) and 15% for ELISA (*n* = 16). These values are consistent with inter-assay *%CV’s* reported for other IgM assays (11–13% [23], <20% [24], 8% [25], 14–21% [26]). Signals from dilution series (1:100, 1:200, 1:400, 1:800) for eight samples tested by FMIA and ELISA were normalized to fall on a single linear response curve for each method (Supplementary Figure S4). The linear ranges were qualitatively comparable for FMIA and ELISA. 

## 4. Discussion and Conclusion

We developed a FMIA platform that allows rapid, multiplexed, and high throughput sample testing and gives analytical results comparable to ELISA. The FMIA provides rapid results (<30 min), requires fewer user steps than ELISA, uses reagents that can be stored in stable dry form, provides multiple assay results (including controls) within a single sample well, and generates visible spots that can be quantified by a camera or scanner [3,4,5]. Table 1 compares features of the FMIA to conventional ELISA; the origin of these features is described below.

**Table 1:** Feature comparison for the flow-through membrane assay (FMIA) and conventional ELISA.

	ELISA	FMIA
Assay time	2.5 h ^a^	30 min
Number of user steps	12 ^a^	5
Substrate	Flat microplate	High surface area membrane
Detection reagent	Two-step enzyme detection	One-step gold detection
Readout method	Microplate reader	camera, or scanner
Targets per well	Single	Multiple
Assay controls	Separate wells	Integrated within each sample well

^a ^For the indirect anti-LPS IgM ELISA used here. Assay time for ELISA is often longer.

First, the membrane provides a surface area that is ~150× greater than the flat plates used in ELISA. The high surface area increases signal density to allow a one-step labeling using gold nanoparticles, rather than the two-step enzymatic amplification used in ELISA. However, the entire surface is not visible to the observer, as it is buried below a highly-scattering surface. Only about the top 25 µm can be observed optically in wetted membranes (data not shown). The gold reagents can be preserved in dry form without refrigeration [4,21], the number of assay steps is reduced, and the results can be read without a plate reader. Here, we used a flatbed scanner to quantify results, but we routinely use a simple webcam to image membranes [3,4,5]. 

Second, the small membrane pores (450 nm average diameter) allow rapid diffusion of analytes and reagents to the binding surface, and controlled flow-through by vacuum allows all sample and reagents to contact the binding surface. In ELISA, slow diffusion to the binding surface leads to long incubation times for several assay steps (*i.e.*, sample capture and labeling). The FMIA essentially eliminates the diffusion limitations; the time required for a molecule to diffuse across a pore is *τ *≈ *r*^2^/*D*, which for a large protein (*D *≈ 1 × 10^−7^ cm^2^/s) and the FMIA membrane pores (*r *= 225 nm), is ~5 ms. Thus, the speed of the FMIA is limited not by diffusion, but by the rate that sample is delivered to the membrane by flow (a controllable parameter) and the inherent binding kinetics for the reagents. Rapid diffusion and controlled flow-through are the primary reasons that the FMIA can be performed in about 30 min compared to 2.5 h for the ELISA protocol used here. 

Third, the membrane can be spotted in a 96-well array with multiple analyte capture spots and integrated control assays within a single well. The indirect assay format uses a single detection reagent to label all assay spots, and analytes can be added by spotting the appropriate capture antigens. In contrast, ELISA plates are typically coated with a single antigen or antibody per well, and assay controls must be performed in separate wells; a failed step within a single well cannot be detected. In the panel tested here, one known positive sample had missing control spots in the FMIA and was identified as a failed test; the sample was re-tested and the results matched ELISA. Without control spots integrated into each sample well, this test would have resulted in a false-negative result for the sample. Further, we found that the patient samples presented a wide range of background levels due to non-specific binding to the FMIA membrane, and un-spotted regions of the membrane within each well allowed background subtraction to account for non-specific binding. The equivalent control was not performed for the ELISA data, but when this control is used it requires running the sample in a separate control well (with no capture molecules). Thus, integrated controls and individual background subtraction used in the FMIA should reduce the risk of false positive results (from “sticky IgM,” which bind nonspecifically to the surface of the blocked membrane), and false negative results (from human error). 

Finally, ELISA results are notoriously difficult to compare between multiple laboratories. This is in part due to the fact that signal amplification is enzyme-based, which is highly sensitive to accuracy of application, reaction time, and temperature. FMIA resolves this by using a gold-nanoparticle-based reporter system, the same that is the basis of lateral flow point-of-care tests.

The FMIA format uses relatively low-cost equipment to perform the assay and read the results, but it requires a method for spotting capture reagents on the assay membranes. The flatbed scanner used as a reader for the FMIA is a less expensive alternative (~$50–300 USD) to an ELISA plate reader (>$5,000 USD). The bead-based IgG depletion method used for the FMIA required a centrifuge (~$2,000 USD), but a centrifuge is typical equipment for separating cells from plasma for either platform. The Bio-Dot vacuum manifold costs ~$500 USD and is designed for reuse indefinitely. The vacuum control was fabricated for less than $90 USD in parts; we used laboratory vacuum, but a common aquarium pump can provide a fine vacuum source. On the other hand, we used an expensive reagent spotting system to prepare FMIA membranes for multi-analyte testing, and this equipment will not be available in most laboratories. Since spotted membranes can be stored without refrigeration when dry, they could be prepared at a central location and shipped as ready-to-use disposables. Alternatively, users could prepare custom assay membranes using modified office printer [27,28,29,30]; however, inexpensive spotting methods may decrease assay performance, and we did not test this option here. 

In summary, we demonstrated that the FMIA is a rapid, multiplexed, high-throughput platform for antibody detection assays and showed that analytical performance compares well to ELISA. We have also developed FMIA tests for different disease analytes including antigens (e.g*.*, malarial *Plasmodium falciparum* histidine-rich protein, *Pf*HRP2 [3,4,5,19] and other IgM antibodies (dengue, measles, spotted fever; unpublished results). In addition, we have integrated the FMIA into a microfluidic card format that performs all sample preparation steps [18], includes on-board dry reagents, and reports multiplexed assay results for point-of-care applications [19]. The 96 well FMIA format reported here has been a useful tool for assay and device development in our lab, and it could be useful for testing in settings where a rapid, high-throughput, multi-analyte method is needed.
